# Foreign

**Published:** 1840-08-08

**Authors:** 


### FOREIGN.

### *On the Gastro-Catarrhal Fever of Peru.* By ARCHIBALD SMTTH, M. D.

[Concluded from page 500.]

*Case* 2.—A middle aged man, of active habits, a respectable station in life, and of European parentage or descent, on the second evening of Carnival, 1836, made a hearty supper on Chiloe ham, cheese, and eggs, &c., and next day, in sport, they threw upon him a bucket of water, and drenched him all over. This wetting was followed by slight febrile symptoms, and he took some simple laxative; to co-operate with which his wife administered not less than ten clysters, under the impression that his indisposition was owing to *empacho* in consequence of the Carnival supper.

Finding, however, all her endeavours unsuccessful, she called to her aid the family doctor, who, in *junta* afterwards assembled, related that, on his first visit, he found the patient with a strong fever; foul tongue; strong pulse; dry skin; much headach; catarrhal symptoms, and difficult respiration. As often as he coughed, he complained of a most acute pain in the head; and the breast on these occasions (to repeat the patient's own words) felt as if a knife were stuck into it.

The doctor, reflecting on the joint import of the symptoms enumerated, thought that the disease fully declared itself as one of an inflammatory nature, and, therefore, the barber was sent for to bleed the patient. Meanwhile, whey was allowed as a lenitive diluent drink. Thus free perspiration was induced, and all the morbid symptoms were quieted for a while. But next morning wandering pains were felt, at one time in the head, at another in the breast, &c., and therefore his physician again ordered him to be bled and to continue the whey. With this arrangement all the parties were contented to remain in a state of expectancy till next morning, conceiving it probable, that the fever would take on the prevailing type of the season,—that is, declare itself a bilious remittent. The patient passed a very bad night, and on the morrow, being the fifth day of his indisposition, the dry skin and tongue, which attended the febrile increment, were preceded by chills and shivering, and the matter expectorated was observed to verge to a sanguineous tint. The family now got alarmed, and a medical friend was called in to meet the family doctor. By the consent of both these professional gentlemen a third venesection was practised; and the patient was put on a ptisan of chicken-water, (*agua de pollo,*) mixed with equal parts of linseed tea, until the doctors should return at the evening visit, further to observe the changing aspect of the case.

At vespers, or after sunset, four medical men, of whom I was one, assembled in junta, when we found the patient in a state of diaphoresis, after having perspired pretty freely since the morning visit of the two physicians; and a small, figured, yellow-coloured stool was shown to the members of the junta. The urine was high coloured; pulse full and strong; tongue a dirty yellow; head pretty easy; no pain on full inspiration; cough much diminished from what it was shortly before; and the expectoration was free, of a watery and frothy appearance, with some thin mucus among it.

The act of respiration, though easy, was attended by a wheezing sound; and by approaching the ear to within a short distance of the chest, there was a distinctly audible gurgling sound, which conveyed the idea of its being emitted, not from the bronchiæ, but from the heart, which palpitated greatly. The whole epigastrium and right hypochondriac region were sensible to pressure, and tender, and the belly felt puffy and full. It would be tedious for the reader to follow the minute details of this gastro-catarrhal fever which terminated happily.

I may, however, remark, that a slight inflammation appeared on the throat, and suddenly passed, as by metastasis, from one side to the other. The head was so much affected during the exacerbations, that though relieved in the intermissions, it was feared lest the excessive tenderness and irritation shown in the brain during these high stages of fever, might be transformed into a state of formal phrenitis; and hence the junta felt it their duty to recommend that the patient should confess himself to his spiritual guide whilst his mind remained entire.

He used the foot-bath, and was once or twice bled from the ankle, by the advice of the junta; and, when the cerebral irritation was considerably lowered, a blister, to produce counter-irritation, was applied to the nape of the neck. The bowels were opened repeatedly and freely by appropriate purgatives, and as the stools, which were dark and copious, fermenting after a short time like yeast, were day after day presented, the good woman, wife to the sick, rejoiced to see her ideas concerning the *empacho* of her husband, now verified to her full conviction.

A spare, cooling, and diluent regimen, was observed throughout; the head was shaved, and evaporating lotions applied to it; and latterly, after the catarrhal symptoms disappeared, pure pine juice was allowed as an antibilious refrigerant. In short, on the ninth day of his illness, and fifth of the medical junta, this patient was declared convalescent, and his usual health was soon recovered.—*Edinburgh Medical and Surgical Journal.*

---

*On the Nervous Fever of Peru.* By ARCHIBALD SMITH, M. D.—Fevers which, in their origin, are attended with high vascular action may, in the course of a few days, assume an adynamic form, and come under the name of the low-nervous, *perniciosas*, or malignant fevers of the Limeno.

In this manner intermittents and remittents are seen to degenerate into the continuous and pernicious; and the unfavourable transition in these instances appears to be less under the influence of common, occasional than of particular constitutional causes. Happily, however, fevers of malignant character (of which I have witnessed more in spring than at other seasons) are comparatively rare on most parts of the coast of Peru, and must be considered as deviations from the more ordinary and milder appearances of the prevailing fevers of the country.

I will here offer a few illustrations of these pernicious fevers, as they are commonly called.

A robust young man, when affected with high fever of only one day's standing, requested the assistance of his medical adviser, who found the pulse strong, full, frequent, and rebounding, with a strong throbbing sensation at the temples; skin dry and very hot; tongue foul, with bright red edges; slight pain at epigastrium on pressure, and irritability at stomach, indicated by nausea or vomiting.

After one or two bleedings, and other ordinary means to mitigate the severity of the attack, in a few days it entirely changed its aspect. The pulse became low and excessively frequent, or small, fluttering, and too rapid to be easily counted; the patient became feeble and tremulous, while the skin continued hot and dry; the fever exhibited irregular exacerbations, rarely terminating in sweat; and headach, drowsiness, or some marked disorder in the head, showed itself more and more, as the disease advanced to its crisis, in the form of salutary sweats. With the help of neutral salts in the morning, and anodynes in the evening; farinaceous diet; and lastly, change of air, the patient ultimately recovered, after a tedious convalescence.

I have seen the bilious remittent after several days' continuance degenerate into the state of the *perniciosa* or malignant. Thus, in the latter end of October, a young gentleman of a stout appearance, and a native of Europe, was taken unwell in Lima with a fever, which soon declared itself a regular remittent. It was attended with tenderness at the epigastrium, furred tongue, headach (increased during the evening exacerbation,) belly loose, the stools being thin and frequent, at first green, and then of the colour of orange-red or yellow. The skin was dry and hot, the pulse strong and frequent, the urine scanty and high coloured, and the patient anxious in his mind.

Blood was twice drawn to the extent of about one pound at each time; he had several warm baths, emollient applications to the abdomen, laxative enemata, sinapisms to the feet when the head appeared much affected, and for food and drink he had arrow-root or thin rice-pap, alternated with whey. A consultation was called, and shaving and cupping the head, &c., determined upon. What particular remedies were afterwards used I cannot say. But after several days had elapsed, this young man became the subject of various medical consultations; and, finally, he sunk so low, that, with great debility and a small, quick pulse, his tongue appeared furred, and of a dirty, dark aspect; lips parched with sordes on the teeth; he had also deafness, and, during the night, stupor and incontinence of urine. He recovered under the influence of an effort of nature. The cupping, which had been early recommended in junta, was not carried into effect under the superintendence of the physician, who was left in special charge of this case. But nature did what the doctor omitted; for, when the case had become nearly hopeless, a burst of epistaxis spontaneously supervened, and rescued the patient from approaching death. This spontaneous emission from the nostrils removed cerebral congestion, restored consciousness, mental health, and the power of the will over the muscular motions of the body. Hence convalescence at once followed the natural crisis by epistaxis. And the event offered this important lesson, very worthy of being borne in mind by those attending cases of a similar kind, that, by timely interference and seasonable local depletion, the happy effort of nature might have been anticipated; and that, if cupping had been resorted to in an earlier stage of the disease, the patient's sufferings might have been shortened, and the imminent peril that threatened his existence easily averted.

In other cases of pernicious fever, the precursory symptoms of excitement may be exceedingly moderate, and the nervous type show itself without any well-marked synocha. Thus I have known a young woman feel as if chilled, "resfriado," and take pectoral orchates with a view to remove the catarrhal symptoms; and the physician who commonly attended her ordered her to be bled, but in very small quantity, under the suspicion of the existence of an obscure peripneumony. When called to visit her I observed that the bowels were costive, and when acted upon by opening medicine, the matter voided was dark and of a highly offensive smell; the pulse was small and extremely frequent, hardly to be counted; the eyes were suffused and red; attention distracted, and the mind confused; the skin dry or partly humid, with partial and transient sweats; great irregularity in the distribution of nervous energy, as indicated by rapid and alternate increments and decrements of fever, and unequal heat of surface, without any apyrexia or interval of fever. With the further progress and termination of this case I am not acquainted; but enough is stated to characterize the type of the fever.

I was called to see a stout young man in Lima, when sinking under a continued fever of several weeks' duration. He was reduced to the deplorable state of lying with his eyes and mouth half open, teeth incrusted with dry sordes, the tongue dry, rough, and brownish; senses heavy and intellect dull, with language occasionally incoherent; the pulse one hundred and twenty, small and fluttering; the belly was costive; and the urine high coloured. Before I saw him in this condition he had been treated by stimulants, tonics, and cordials; but, by giving him two grains of calomel every four hours in the form of pill, before he had taken thirty grains, the gums became mildly affected, and in three days he was convalescent. As soon as the mouth was touched by the remedy, his bowels were freely evacuated by a dose of salts and senna, when the depositions carried off were as dark as ink, and intolerably fœtid. After this the disease assumed quite a different aspect; profuse perspiration followed, the circulation became equalized, and the young man had a refreshing sleep, from which he awoke with a clear and collected mind. Henceforth he had a favourable convalescence, and recovered perfectly.

Fevers beginning with symptoms of high excitement, and ending in the depressing manner which I have briefly described, are frequently known to arise, on the sandy plains of the coast, from exposure to intense solar heat; and then the head is the part first complained of, and violent fever of a pernicious tendency ensues. I have known a young Indian thus attacked when bathing himself in a deep pool on a very hot day, with all except his head in the water. He came out from the bath with a violent headach; and immediately covered over his head with a poultice of wet mud, which, he said, was considered a good remedy in his native place. The fever appeared with high vascular excitement, for which he was bled, and the bleeding relieved without removing the headach. He resided a few miles out of town, and had not the advantage of regular medical assistance, though he had the good fortune of residing in the hospitable mansion of a charitable gentleman, who said he always cured the poor Indians about him by active English remedies, as salts and blue pill, but that the rich Indian who went to Lima to be cured on the *spectative* plan commonly died. I saw this poor Indian afterwards in a state of convalescence, and he recovered slowly. When at the worst his skin was reported to have been hot and dry, the pulse extremely low, body quite tremulous, head ached continually, and the lower belly was tender on pressure, while the tongue looked perfectly clean.

I have witnessed the case of a praiseworthy ecclesiastic of Lima, who, though never accustomed to go out of town, had a painful inducement to visit Chorrillos, (three leagues from Lima,) when he heard that the curate, whose assistant he was, died at this watering-place. The weather being very sunny, and the air of the close conveyance in which he was seated being very hot, he, unaccustomed to exercise, felt the exertion of going and coming the three leagues too much for him. He was also distressed in mind for the death of his friend: and, it was said, he inhaled some deleterious exhalation from the dead body confined in a shut up apartment, which he opened and entered without any precaution or previous ventilation. These causes conjointly were assigned for the fever, which appeared in its origin with symptoms of synocha, but terminated in a rapid failure of vital power, proving fatal on the fifth day.

I was only called to see him on the last day of his malady, when I found his pulse frequent, fluttering, and yielding, or retiring under the gentlest pressure of the finger; the tongue perfectly white, which he showed when desired, though he did not speak; and he appeared to be, when left undisturbed, in a somewhat lethargic state. In this drowsy and sunk condition, his attendants said he had only been since the day before. A stool was exhibited, which consisted of hard fæces, like hazel-nuts or filberts in size and colour. No opening medicine, except the customary clysters, had been administered. From the commencement of the attack, he had headach and tenderness at the epigastrium. He was twice bled in the early part of his sufferings; but his head was not shaved; he had a succession of sheep or pig cawls applied as an emollient to the abdomen; he drank cooling ptisans and "frescos con nieve," or refrigerating beverages with ice, which were not suspended till the fourth day of the disease, viz. on the day preceding its fatal termination.

With regard to the treatment adopted by a native practitioner in this case, I may be allowed to express my disapprobation, and observe that, if, after the first or even second general bleeding, (though in fevers of adynamic tendency depletion should be used with great circumspection,) the bowels had been opened freely,—the head shaved and kept cool by proper lotions; or, in the event of this not proving sufficient to relieve the head, had cupping-glasses been applied to the nape of the neck, and a good blister to the epigastrium, such means would at least have afforded the patient a better chance of recovering than what fell to his lot.

The use of iced-drinks in this fatal case was not appropriate practice under the actual circumstances; for from the tenderness at the epigastrium it may be supposed that the stomach was not free from acute inflammation; and there was ocular evidence from the appearance of the alvine discharges, that the condition of the bowels had not received proper attention. If iced-drinks could have been at all admissible in such a case, they could only be so, as in other instances of daily occurrence in which they are had recourse to, after the bowels had been suitably prepared for them by clearing out their irritating contents. There appears no good reason for having omitted the shaving of the head of this ecclesiastic; but it may be observed, that to this practice the inhabitants of Lima are very averse. In a case of repeated consultation, in which it was deemed necessary for a young woman's safety to apply a blister to the occiput, I have known some native physicians so sympathizing and sensitive, as only to allow the barber to expose a surface about the circumference of a dollar, or little more than what every friar shaves on the vertex or crown of his head. But, however scrupulous native practitioners are in despoiling the Limenas of that natural ornament which they all prize so much, as never to crop from their childhood upwards, yet life is surely of more value than the handsomest tresses of the female patient.

It is an important feature in the pernicious fevers of Lima, that whether the affection of the head be primary, or, as more usually happens, secondary, or apparently coeval with gastric disorder, we are seldom called to any case of this nature, especially when of some days' duration, in which we do not find a morbid degree of irritability of the stomach, demanding the utmost attention, both as respects diet and medicine. For the rapid sinking of the general strength, or vital powers, may be in these cases, observed to be connected with a morbid condition of the gastric organs.

It should further be remembered, that the convalescent from one of those attacks requires so nice an adaptation of the nutriment he takes, to the impaired strength of the digestive powers, that he may become delirious at night by the indigestion of a soup, somewhat too rich or gelatinous, taken in the middle of the day; and relapses often occur when all danger is thought to be over, because the attendants are only solicitous to re-establish by generous diet the strength of the patient. In consequence of a sudden transition from farinaceous and low diet, to eating the breast or wing of a chicken, or any sort of animal food, patients from being convalescent are seen to fall into a state of stupor, from which they are perhaps so fortunate as to be roused again by the operation of an effective purgative, followed by a blister placed on the region of the stomach. And in some such cases, I have known the healthy functions of the brain recovered at the expense of a general bleeding; which, however, can rarely be so eligible as local depletion in such instances, as it will be commonly found, that the persons affected have already been much debilitated by previous venesection and protracted disease.

From topical emission of blood at the occiput or nape of the neck, I have had the pleasure to witness the most beneficial effects, when *tercianas de cabeza*, formerly alluded to, or more or less perfectly developed intermittents with a particular determination of blood to the head, hurried on to assume what may be called a typhoid or low nervous type.

Camphor, musk, and all such remedies, though frequently used in native practice, I have never known to do good when tried in the pernicious or malignant fevers of the country; on the contrary, they do harm; and in almost every case wherein I have known them to be administered, gum-water, the alleged specific for gastro-enteritis, would be a far better remedy. I once attended in consultation, a young man, in whom a common *terciana*, to the best of my recollection, had degenerated into a fever of a low nervous aspect, which the consultation was called in to remove. The fever never left the youth, whose bowels were costive, skin dry, mouth parched, and pulse low, but much accelerated; his spirits were dejected, and petechiæ appeared to a considerable extent on his body. Here the treatment adopted was to give the infusion of Peruvian bark, with a small quantity of sulphate of magnesia several times in the course of the day, and by this means the bowels were kept open without causing any such excessive evacuations as could further debilitate the weakly patient. Cold water, with the juice of the pine-apple or some other cooling fruit added to it, was drank after each dose of the bark in particular; and generally, or at any time, he was permitted to partake of the same grateful drink when he had urgent thirst; always recollecting this exception, that, if there should be any diaphoresis, or indications of more free perspiration, cold drink was absolutely forbid. His aliment consisted of pap, made from rice and bread, seasoned with a vegetable acid. Under such treatment the young man recovered.

Fevers of adynamic termination are sometimes accompanied with parotid swellings; but not frequently, so far as I had an opportunity of witnessing.

In consultation, I have seen one memorable instance where, at the close of the fatal disease, the symptoms were those of a genuine *typhus*

*gravior.* The case alluded to was that of an accomplished and fashionable young lady,* whose death was deeply lamented among those of her own high rank in society.

But it is to be observed, that though such aggravated cases of what might be called sporadic typhus are now and then known to occur in Lima, and in the warm valleys adjacent, or along other parts of the Peruvian coast, yet, we never find that these malignant fevers spread from house to house, or from individual to individual. Never are physicians or attendants on the sick affected with it from immediate contact with those who suffer from it; and the stranger who first witnesses a case of this sort in Peru, after having been familiar with typhus in Europe, is apt to conceive that danger is to be apprehended from contagion, and he directs his measures accordingly. But the native practitioner laughs at his imaginary fears, and assures him his ideas are erroneous, and that experience will satisfy him that the pernicious or malignant fever of their happy clime is never contagious.

To this statement, however, there remains to be mentioned a singular exception.

Dr. J. M. Valdes of Lima, in a pamphlet on the Epidemic which prevailed in his native city during the siege it underwent in the year 1821, states at page 23, where he treats of the continued bilious fevers that had degenerated into putrid or malignant,—that is, fevers in which there was observed a sudden prostration of strength without manifest cause,—that this fever was contagious in the hospitals, and in the houses where many sick were assembled together. And at page 27 of the same valuable treatise, he remarks, that the exciting causes of this epidemic were assuredly consequences of the war, which deprived the inhabitants of the salutary use of ice, good bread, and wholesome food, and which kept their minds in terror for the loss of property and life. This is the only notice that, during my residence in Peru, I had of the contagious power of the putrid, or what is usually called the pernicious or malignant fever on the coast. And supposing the doctor quite correct in his observation of the fact, as stated by him, it is obvious that the anomaly is one peculiar to those revolutionary times and circumstances, and not a fact of ordinary occurrence in the practice of the Limese physician, who, as I have mentioned, laughs at the very idea of contagion.

*Ibid.*

---

*On the Exanthemata of Peru.* By ALEXANDER SMITH, M. D.

Among the cutaneous diseases prevalent in Lima, may be enumerated, first, the principal febrile eruptions; and in the second place, various exanthematous affections unaccompanied with fever.

A. *Febrile Eruptions.*—1. Miliary eruptions, vulgarly called sarpullidos; neither infectious nor always attended with fever;* 2. Variola; 3. Varicella; 4. Rubeola; 5. Roseola; 6. Acute Erythema, unusually symptomatic of gastric disorder; 7. Scarlatina; 8. Urticaria; 9. Purpura; 10. Erysipelas; 11. Furuncular tumours called *chupones*, frequently attended with smart fever, and most acutely painful; 12. Anthrax, or carbuncle, which appears in the loins, back, or nape of the neck, and is prevalent among the bronzed and negro classes; 13. *Grano de peste*, or a malignant furunculous pustule, which usually appears on the arms and face.

On the above catalogue of cutaneous affections, it would be superfluous to comment in detail; but a few observations may be offered on some of the diseases enumerated.

1. *Variola.* This disease, so fatal in some other parts of Peru, is in Lima a comparatively mild disorder. Indeed, vaccination has become so general in the capital, that there, at least, through the diligence of Dr. Figueroa, the small-pox is nearly exterminated. In private practice I have only seen one or two cases of the disease; and Dr. Herrera, Physician to the Hospital de Incurables, where patients affected with small-pox are usually sent, informed me in October, 1835, that during two years he had then been in charge of the hospital, he had only lost one patient of "*Viruela*," or variola, and mentioned that this disease scarcely ever appeared in a malignant form among them. They are chiefly slaves from the estates surrounding the capital, who are in the present day received into the Hospital de Incurables; not, however, as incurable, as the name of this institution would imply, but to be cured, and that at the easiest and cheapest rate.

2. *Rubeola.* This is an exceedingly prevalent but mild disease, which occurs at all seasons.

In the same family the eight following cases presented themselves to my observation in the spring of 1835, when measles prevailed in town; and as they show how the disease, if allowed to have been of the same nature in all those instances, may be modified in individual constitutions, and persons of different race or cast, when exposed to the same source of contagion, I shall notice them separately.

*a.* A boy of Spanish descent, and about 9 years of age, was the first of the family affected. On him the eruption was general; the fever very moderate, and there were no catarrhal symptoms.

*b.* A young gentleman, (brother to the above) 14 years of age, was affected with mild *cynanche tonsillaris*, but no efflorescence appeared on the skin.

*c.* A Mestiza girl, 10 years of age, was affected with a sense of oppression at the scro-

---

* Miss Taramona.

* Under the term "Sarpullidos" may be included the Lichen Tropicus, or prickly heat.

biculus cordis, and moderate fever (during which she vomited a "lombriz," or long round worm,) which preceded the cutaneous eruption. This patient had slight sore throat, which, on inspection, presented a red and irritated appearance, but without tumefaction.

*d.* A Negrita, or negress, 12 years old, was affected, in like manner, with the Mestiza, excepting the accident of the lombriz.

*e.* A Cholito, or little Indian boy, had a mild fever and light eruption, with a slight uneasiness at the throat, accompanied with unusual redness of the fauces.

*f.* The coachman, a Zambo lad, had a very copious and general eruption, and a strong precursory fever, aggravated probably by having persisted, for the first days of his illness, in taking hot mirasol, or red agi-pepper, in his soup.

*g.* A young Zamba girl, 11 or 12 years of age, suffered a very mild attack, and had but a slight eruption. She, like the coachman, never complained of sore throat. It is to be observed, that two or three days before this girl's febrile disorder appeared, her bowels had been acted upon pretty freely by clysters,—a circumstance which probably conduced to the mildness of the measles which followed.

*h.* A Zamba, about 17 or 18 years of age, was the last person in the family affected with this disease, and in her case the fever ran high, attended with oppressed breathing and catarrhal symptoms. She had a perfectly formed cynanche tonsillaris, and with it a profuse eruption on the skin; and as the eruption retired and the throat recovered, she was seized with cynanche parotidœa, but she soon got quite well.

This girl, some years before, had suffered from hæmoptysis, and was suspected of being in a state of confirmed decline; but long before this attack of measles, she had been considered quite free of all these old complaints.

In the above eight cases, which occurred, as mentioned, in the same family or household, the precursory fever varied from three to six days; and the shortest fever was in the case of the youngest, or the first of those attacked as described. All the cases occurred in the course of about twenty-eight days, the one patient recovering as another fell ill; and, occasionally, several of these individuals were confined together in the same apartment at the same time.

3. *Roseola.* This affection I would include in those transitory efflorescences indiscriminately called "*brotes*," and which are ushered in by some restlessness and acceleration of the pulse. The general denomination *brote*, or rash, is applied to those varieties of cutaneous appearances so common in infancy and childhood, for which the natives have as yet adopted no specific names. By dieting or changing the nurse, by open bowels, general cleanliness, and daily bathing, these affections are commonly rendered harmless; and little professional curiosity or interest is displayed in the ordinary management of such exanthemata.

4. *Scarlatina.* This disease is happily not so frequent as rubeola, yet it is well known in Lima,—principally in the form of *scarlatina simplex*, or *anginosa.*

In the year 1832, I was called in consultation (not many hours before the patient died,) to assist a Chilian gentleman affected with a very malignant case of angina, or ulcerated sore throat, which was made the subject of proto-medical inquiry at the time. A few days after his arrival in Lima, he died of this malignant sore throat, the seeds of which he must have carried with him from his native country; for he was heard to say that he had come away from home, "huyendo del peste," or flying from the plague of a scarlet fever that then raged in Chili.

In this case there was no distinct exanthematous affection of the skin; but we may be allowed to infer that the inflammation of the throat and mucous linings of the first passages was so concentrated as to have prevented the appearance of an external efflorescence or eruption, which would probably have appeared had not the patient been early destroyed by gangrene. That this fatal case was essentially of the nature of the Chili epidemic, or scarlatina, is verified by the fact that a military friend who visited him in his last illness, as well as his nurse, were immediately after seized with scarlet fever in a mild form; but precautions were now taken, and the contagion was not allowed to spread.

I am of opinion, that if yellow fever (unknown on the shores of Peru,) were ever to be imported as the scarlatina had been, it would not propagate itself by contagion, provided the public precaution were taken to place the sick at a distance from one another, and in well ventilated situations, so as not to form a focus of infection, as happens in confined situations, such as ships and prisons. And further, by being conveyed one day's journey from the coast to the inland country, the infected could be at once (even in the most sultry months of summer) transported into sufficiently elevated localities, where the change of air and climate should infallibly annihilate the contagious power of this very formidable malady, so familiar to the inhabitants of some parts of the eastern shores of Spanish America.

But to return from this digression, I may here remark that there is a form of angina, which appears to be in some degree akin to scarlatina, unaccompanied with any cutaneous eruption, and of frequent occurrence in Lima. It is sometimes preceded by rigors and fever, at other times the alteration in the pulse is very little, but there is a soreness in the throat at the time of swallowing. The tonsils appear very little, if at all, swollen or elevated, though they may be slightly and superficially ulcerated, or have an aphthous, and grayish or whitish aspect.*

This affection of the throat I have seen various persons, chiefly adults, affected with, at or about the same time: but I could not ascertain that it ever was contagious, though I have seen it occur when scarlatina was known to be in the city.

In several instances, I met with this kind of angina, accompanied with a low nervous fever, and great prostration of strength. Where the affection of the throat was slight, opening medicine and cooling tamarind drinks were sufficient for the cure. In severer cases I was accustomed to prescribe, with satisfactory effects, an emetic of ipecacuanha, and afterwards administer the infusion of bark, with a small quantity of a neutral salt, just enough to keep the bowels easy, without producing debilitating evacuations.

I have known a young lady in Lima affected with a malignant sore throat, without any cutaneous eruption, whose ailment (considered to be a variety of scarlatina) was greatly aggravated by the musk and hot regimen prescribed her, by some young medical gentleman, who earnestly resorted to the application of stimulants, as they saw her strength sink under an adynamic fever. I was called to see this person, and suggested the propriety of leaving off the stimulating plan of cure. The consequence was, that a dose of sulphate of magnesia to relieve the bowels, followed by the bark infusion, restored her readily to health, after her situation had excited great concern.

5. *Purpura.* It may be noticed generally, that the purpura simplex, or small petechial like flea-bites, the *Porphyra pulicosa* of Dr. Good, is a very common appearance in Lima, especially among women and children, in chronic febrile diseases, when those affected happen to be laid up in ill-ventilated apartments, or residing in narrow lanes on the banks of the Rimac, or in the sultry, confined apartments attached to little retail shops in nearly all the principal commercial streets of the city.

But the *purpura hæmorrhagica* is seen on the coast to affect, with remarkable frequency, Indians of either sex, recently arrived from the interior of the country; though the disease is by no means confined exclusively to such persons.

Boys newly got from the inland valleys, who have lived chiefly on fruit and vegetables, and are in the capital intended to be brought up to serve at the tables of the wealthy, where they eat animal food, and live on a more generous diet than they have been accustomed to from their childhood, are those in whom I have witnessed the worst examples of the purpura hæmorrhagica. The affection in those instances came on without any premonitory symptoms of illness, while the boys were to all appearance, but a day before the attack, perfectly well.

This disease, when it occurs in the form of a continued oozing from swollen gums, with small frequent pulse, and an appearance of dark blood in the stools, together with innumerable small livid spots on the skin, may be cured readily by a mild efficient purgative, such as an infusion of senna and rheubarb, with the addition of a neutral salt, followed up by small doses of sulphate of quinine dissolved in water sharpened with sulphuric acid. But in other instances, the hæmorrhage from the nose and mouth, &c., is so active, and kept up by such strong vascular action, indicated by a full, hard, and frequent pulse, that it can only be subdued (and that not unoften but for a short time) by the use of the lancet; after which, purgatives and acidulated drinks may be of efficacy.

6. *Grano de Peste.* The following account, which I think well calculated to convey a correct idea of this disease, was verbally given me, in March, 1836, by a respectable woman in the part of the suburbs called *Malambo*, only a few days after she had recovered from an attack which she described in the following terms: "It began on the left arm, like a mosquito bite, being merely a small red speck, with a sort of a minute vesicle in the centre. It went on rapidly increasing with intense fever; and in four days the whole arm had swollen so as to appear ready to burst, and the swelling reached the left breast. The spot, which at first looked only like a mosquito bite, now became a hard grain or boil, entirely black, and surrounded with a red and indurated base. The little vesicle in the centre, opened a small mouth, or orifice, through which there was no discharge. At the end of eight days, an incision was made into the grain, but nothing escaped from it, except a thin ichorous discharge, or 'aguadija.' The grain attained at last the size of a black grape, but it was flat at the top, (*chato*) and of a hard consistence until opened. It was cured by emollient fomentations, and two bleedings ordered by the doctor."

We are to understand, as a matter of course, and therefore taken for granted in this woman's description of her own case, that occasional enemata and acidulated refrigerant drinks were here used according to custom, and at no time lost sight of during the progress of the disease or treatment. I may further remark, that, in its commencement, the Grano de Peste, which is not contagious, is usually attended with fever of inflammatory character, and, if not well managed from the beginning, it soon degenerates into an adynamic form, very often

* The aphthous sore throat of young children is a very common affection, as may be easily conceived, since the alimentary organs are very commonly disordered. To remove this affection, the natives use various acidulated syrups, all of them quite inferior in their effects, as local applications, to the common mixture of borax with honey.

ending in death. Hence the practical importance of checking, in proper time, by venesection, that high excitement, and strong vascular action, with which the disease commences in young and robust subjects, and which naturally leads, if not controlled by art, to a succeeding state of prostration of strength and exhaustion of vital power.

B. *Exanthematous Affections unaccompanied with Fever.*—Of the Exanthemata unattended with fever, and of common occurrence in Lima, but of which I have preserved no written descriptions, I will here enumerate such as clearly present themselves to my recollection, without desiring to have it imagined, that the eumeration, though correct as far as it goes, is by any means a complete list of such cutaneous affections.

1. *Ecchymomatous discolorations.* These are called "cardinales," by the natives, and appear in the form of black, blue, and livid marks, which are of longer or shorter continuance, arising spontaneously, and, especially in fair-skinned females, and feeble children, without external bruise or injury. After ague-fits, dark marks of this kind are sometimes observed round the eyes; but these extravasations quickly disappear without medical interference.

2. *Pityriasis versicolor.* This discoloration not unfrequently occurs among persons of white and delicate skin, particularly Europeans, in whom I have seen it appear in brownish yellow patches about the neck or abdomen. I have known it disappear by removal to a cool climate.

3. *Leucopathia.* This is a disease which may be observed in negroes, mulattoes, and other dark races, in whom the skin, especially about the face and hands, undergoes a partial change from its natural dusky colour to white, presenting a curious spotted appearance.

4. *Jaundiced yellowness of eyes and skin.* We often see this appearance as symptomatic of chronic liver disease.

6. *Prurigo* is a common disorder in warm weather. There is sometimes complained of, a local pruritus of the genitals, which is intensely annoying and difficult of cure.

8. *Caspas.* This is the native name for slight furfuraceous affections, or desquamations of the skin, very common among the inhabitants of the coast. Sea-bathing is held to be a good remedy for mild attacks of this disorder; but sometimes these may be of syphilitic origin, and therefore require to be considered carefully, and treated judiciously, as circumstances may suggest. I may mention that those affected with syphilitic eruptions are usually very sober in their habits; for even women of the town in Lima, are strangers to the common English vice of inebriety.

9. *Psoriasis.* This disease is not an unfrequent one; more particularly that variety of it called *P. palmaris.*

10. *Lepra* and *Ichthyosis* are both frequent and inveterate affections among the dusky and slave population.

11. *Ringworm* (*Impetigo figurata*) is a frequent and troublesome affection, which seems very generally to depend on constitutional causes; but when not old and unusually inveterate, it commonly yields to cleanliness, well regulated bowels, and the local application of the mild citron ointment.

12. *Scabies or Itch.* This disorder is divided by the vulgar into two sorts; the one, in which the pustules are larger, they call "Carracha Sierrana," or the itch of the interior or hill-land; and the other, which is the commonest variety on the coast, they call "carracha fina," in Lima—in consideration, we suppose, of the smaller sized pustules in this infectious disorder, which they attempt to cure by baths and general bleedings, &c., having a great prejudice against sulphur, which is the well known and most certain remedy.

14. *Pasas or Favi.* The infectious disease to which the Limenos apply the first of these names, consists of soft and painful excrescences, appearing between the nates, or on the piliform parts in the vicinity of the anus. Such of these excrescences as I have had opportunity to see, were of the size of dried grapes, as the appropriate term *pasas*, which means raisins, at once suggests; and like raisins, they are flattened on the surface. They have also irregular edges, and emit some discharge. The local remedy consists in the application of a proper escharotic, as the sulph. cupri, or blue stone, followed by a bread poultice, rest, and cleanliness. Constitutional treatment I have not known them require; though it will always be convenient, should it only be from the usual locality of the disease, that the bowels be kept quite open and easy.

15. *Lupus.* I have seen two instances of a lupus or corroding ulcer on the forehead. Both cases occurred in females; the one middle-aged, and the other far in the decline of life. The former example, which was somewhat more superficial than the latter, healed under the use of aperients and the local application of the citrine ointment, well diluted; the latter appeared to be incurable; for arsenical and other remedies, usually recommended in books, were resorted to without any improvement in the appearance of the sore, which was a deep ulcer, gnawing, and destroying the common integuments and fascia on the forehead, and between the brows. Affections of this kind are not rare, as I am credibly informed, in different parts of the interior, where they are known under the name of "uta;" a word used by the natives to denote an insect, which they suppose to be the cause of the lupous ulcer, to which they therefore transfer the name of the insect.

16. *Elephantiasis.* This term is confined in Peru to a large tumefaction in the leg, which becomes overgrown, clumsy, and through time quite rough on the surface. As far as I have seen, it only affects the negroes, or other dusky persons of African descent. I do not recollect to have seen more than one instance of it in the warm valleys of the interior, and it is possible that this case may have originated on the coast. I witnessed a recent case of it in a young Zambo, who was brought from the vale of Caneti to the house of his master in Lima. When on the sugar estate in Caneti, he was employed as *rigador*, or in irrigating the plantations; and he referred the origin of his swelled leg to his being so much employed in the wet, which few negroes are fond of. He was now made to change his occupation, and to pass from the wet labour of the field to the dry service of the kitchen; and when I last saw him, his leg, though of a large size, had not yet acquired the usual asperity of surface characteristic of elephantiasis in its perfect state. I would be disposed to believe that the tormenting insects named pigues, or chigres, which abound to excess in the slave-barracks or galpon, on the coast, and are known to commit extensive ravages in the feet and toes of the slaves, contribute not a little to induce the great thickening and roughness of the integuments, observable in the elephantiasis of that country.

I should also mention that the Guinea-worm is said to appear occasionally, emerging from under the cutaneous tissues of the agricultural slaves on the coast; but, as I never witnessed a case of this nature, I can say nothing on the subject from personal observation.

*Ibid.*

---

*On the Simple Gastric Fever of Peru.* By A. Smith, M. D.—There is a mild kind of fever which seems to be immediately connected with a morbid condition of the mucous membrane of the digestive passages, and receives the name of gastric fever. It is so frequently met with during the bathing season or summer months in Lima, as to deserve a word in passing.

It is marked by anorexia or disrelish for food; a perfectly white and smooth tongue; some thirst, and a desire for cooling drinks; moderate acceleration of pulse without distinct intermission of fever; almost incessant perspiration, which is eminently favoured by the warmth of the weather. The patient is not so ill as to be confined to bed, but feels listless, and likes the ease of the hammoc or sofa; and usually refers the origin of the ailment to cold, caught probably in the bath, or to cold and some irregularity of diet conjoined, as the result of late evening parties.

This gentle fever the women often manage without the assistance of the faculty, by the administration of the mildest injections;—a light farinaceous diet with vegetable acids, and frescos or cold drinks, from which they are not at all cautious to take the chill, though by this neglect they are apt to check a salutary perspiration, and thereby increase the disorder.—*Ib.*

*Some Observations on the Mode of Union of Fractured Bones.* By R. H. Meade, Esq., Lecturer at the Middlesex Hospital.—This paper, which is one of a series upon the subject, intended by the author to be brought before the society, contains the details of several experiments conducted on rabbits and guinea-pigs, with a view of determining the precise mode of union in fractures of the flat bones, the scapula being here selected for the purpose as the most easily fractured. The animals chosen were of different ages, that it might be ascertained whether age exerted any material influence on the reparative process, and care was taken to inflict as little injury as possible on the soft parts. Preparations and drawings of the parts were exhibited, in illustration of the narrative. From these experiments, (nine in number,) and many others of a similar kind performed on the scapula, the author infers that union is accomplished in the thick part of the scapula exactly, as it is in the cylindrical bones, viz., blood is effused into the different tissues surrounding the fractured part; that this blood is next absorbed, and coagulated lymph deposited in the substances of the muscles, and in the neighboring cellular tissue, so as to form them into a solid gelatinous mass. The periosteum which has been ruptured, is separated from the fractured edges, inflamed and thickened, and lymph is effused between the fragments themselves. At a later period, the external mass decreases in size, the muscles return to their natural texture, and a firm layer of cartilaginous matter surrounds the fractured spot, with which the periosteum is so completely blended, that it is difficult to say whether it is external to, or beneath it. This callus adheres firmly to the surfaces of the bones, and dips down between the fragments. Ossification then takes place, by the deposition of earthy particles in the cartilaginous matter, which particles are deposited irregularly through all parts of the provisional callus at the same time, and do not appear first at that part which is next to the surface of the bone.

The process of union of fractures in the flat part of the scapula differs somewhat from the process here described. In speaking of the part which the periosteum plays in the production of the callus, the author observes, that where two fractured portions of a flat bone overlap each other, and the edges which are in contact are denuded of their periosteum, union takes place between the opposed surfaces through the medium of fibro-cartilaginous substance becoming osseous, which possesses all the characters of the common external or provisional callus. This matter must here arise from the surfaces of the bone itself; and this fact shows that the provisional callus may be generated independently of the periosteum.—*Trans. of the Royal Medical and Chirurgical Society.*

*Lon. Med. Gaz.*

